# Design Strategy and Application of Deep Eutectic Solvents for Green Synthesis of Nanomaterials

**DOI:** 10.3390/nano13071164

**Published:** 2023-03-24

**Authors:** Nguyen Nhat Nam, Hoang Dang Khoa Do, Kieu The Loan Trinh, Nae Yoon Lee

**Affiliations:** 1 Biotechnology Center, School of Agriculture and Aquaculture, Tra Vinh University, Tra Vinh City 87000, Vietnam; 2 NTT Hi-Tech Institute, Nguyen Tat Thanh University, Ward 13, District 04, Ho Chi Minh City 700000, Vietnam; 3 Department of BioNano Technology, Gachon University, 1342 Seongnam-Daero, Sujeong-Gu, Seongnam-Si 13120, Republic of Korea

**Keywords:** deep eutectic solvent, nanostructure fabrication, nanofluid, green solvent, electrodeposition

## Abstract

The first report of deep eutectic solvents (DESs) was released in 2003 and was identified as a new member of ionic liquid (IL), involving innovative chemical and physical characteristics. Using green solvent technology concerning economical, practical, and environmental aspects, DESs open the window for sustainable development of nanomaterial fabrication. The DESs assist in different fabrication processes and design nanostructures with specific morphology and properties by tunable reaction conditions. Using DESs in synthesis reactions can reduce the required high temperature and pressure conditions for decreasing energy consumption and the risk of environmental contamination. This review paper provides the recent applications and advances in the design strategy of DESs for the green synthesis of nanomaterials. The strategy and application of DESs in wet-chemical processes, nanosize reticular material fabrication, electrodeposition/electrochemical synthesis of nanostructures, electroless deposition, DESs based nano-catalytic and nanofluidic systems are discussed and highlighted in this review.

## 1. Introduction

**Figure 1:**
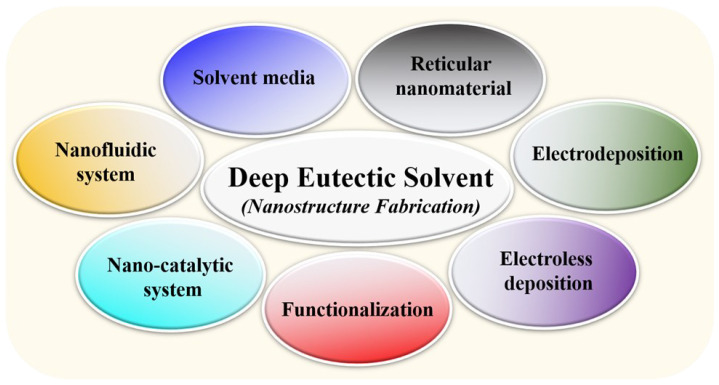
The applications of deep eutectic solvents (DESs) for nanomaterial fabrications.

The distinct properties of solvents contribute significantly to research outcomes and processes in various industrial areas [1,2,3]. Solvents assist in making optimal media for chemical reactions and participate in the different chemical processes. However, releasing solvents after being used has caused harm to the environment and human health [4,5]. Therefore, different methods have been developed to evaluate the risk of solvent to the environment [6,7,8,9]. For example, the conductor-like screening model for realistic solvation (COSMO-RS) quantum chemical computation has been employed to screen chemical reaction solvents [10]. Additionally, the industrial processes based on solvents were evaluated by the environmental index (IEI) [9]. The harmful outcomes of solvent waste resulted in utilizing new environment-friendly and biodegradable solvents. Using solvents in organic chemistry revealed using different solvents for organic synthesis [11]. Nature has different solvents that can be used for chemical reactions and processes [12]. The ingredients for making bio-solvents included animal fat, fruit peels, sugarcane, corn, and wood, which produce ion liquids, alkanes, aromatics phenolics, esters, furans, and ethers [13]. These bio-solvents exhibit properties similar to petroleum-based solvents but are biodegradable and recyclable. Consequently, there was a trend of screening for green solvents to reduce the environmental damage caused by hazardous solvents [14]. Due to their unique advantages and characteristics, such as biodegradability and recyclable nature, green solvents are widely used in several industries, including pharmacy and food processing [15,16]. The discovery of new green solvents has revealed new candidates for different substrates. For example, triazolium-based ionic liquids have been proposed for breaking up the molecular connection of cellulose and can assist with using cellulose-originated ingredients [17]. A notable finding was DESs, which were first announced by Abbott et al. in 2003 [18]. DESs, a famous IL, exhibited features of phase behaviors and physical properties in other ILs [19,20]. Generally, DESs, which are prepared by combining two or three biodegradable components of hydrogen bond acceptors (HBAs) and hydrogen bond donors (HBDs), can be considered green solvents [21,22]. Because of the characteristics of versatility, nontoxicity, and biodegradability, DESs have been utilized in different fields [23]. In the biological field, DESs have been used to analyze biological matrices regarding clinical and toxicological objects [24]. DESs effectively extract biomolecules (i.e., proteins, carbohydrates, and lipids) [25]. Previously, reviews on DESs provided essential information and general applications [26,27]. DESs effectively produced novel and refined materials (i.e., plasmonic metal nanoparticles) [28,29]. In this review, applications of DESs for nanomaterial fabrication were summarized, including the areas of solvent media, reticular nanomaterials, electrodeposition, electroless deposition, functionalization, nano-catalytic and nanofluidic systems (Figure 1). This specific review provides the current status of the applications of DESs in the design and fabrication of nanomaterials.

## 2. Fundamental of DES

The combination of HBAs and HBDs results in the formation of a type of green solvent called deep eutectic solvent or DES [30]. The number of HBA and HBD can be altered with respect to the different classes of DESs. Typically, an approximate ratio of quaternary ammonium salts with metal salts is used to make DESs.

Five types of DESs have been characterized based on the components of HBAs and HBDs. In I, II, III, and IV types, HBAs contain a halide anion and a quaternary ammonium cation (mainly choline chloride was used). However, HBDs are different among the different types. For example, type I DESs have metal chloride as HBD, whereas hydrated metal chloride and organic molecules are present in types II and III. In type IV, metal halides and urea act as HBDs. The recently developed type V comprises natural resources for HBAs and HBDs, such as acetic acid, caffeine, DL-Menthol, lauric acid, pyruvic acid, tetracycline, and tryptophan [31]. The hydrophilic DESs have been classified into types I and II. In contrast, hydrophobic DESs were categorized into types III and IV [32]. A new class of DESs has been described as non-ionic solvents of which hydrogen bonding is especially predominant [21]. This new type V has a general formula as RZ + RP, where Z = CONH_2_, COOH, OH and P = C_6_H_4_OH, CO, NH_2_. DESs generally have highly tunable chemical and physical properties. The properties of DESs typically depend on the nature of the HBA/HBD combination, molar ratio, water content, and temperature [27].

The wide applications of DESs in nanotechnology fabrication can be considered by their relatively high conductivity, low volatility, and high thermal stability. The properties of DESs could be actively driven to use them as a green solvent in chemical processes. The structure of HBDs and hydrogen bonding proton transfer mechanism has been reported to determine the properties of DESs. Different HBDs, including urea, polyhydric acid, polyhydric alcohols, and saccharides, have been explored [33]. Hydrogen bond interactions activated the carbonyl and guanidine groups. Some HBDs of 1,2-propanediol and urea established a strong intermolecular account for the extraction efficiency. The designable mixtures of HBAs and HBDs resulted in different features of DESs [34]. A new liquid phase can be produced from hydrogen bonding based on a eutectic mixture and self-associate originating from HBAs and HBDs. The capability of providing electrons and protons of compounds that have high affinity to DESs enabled various dissolution properties [20]. Another attractive aspect of DESs is the capability of producing various eutectic mixtures that include two non-faceted phases. These mixtures trigger the formation of regular particles that exhibit continuous edgewise growth in melts [32]. Additionally, the melting point of eutectic mixtures is lower than its components due to the effect of hydrogen bonding on the self-assembling capacity of DESs.

The hydrophilicity and hydrophobicity properties of DESs can be considered. The hydrophilic and hydrophobic DESs have been compared for sulfonamide pretreat determination [35]. This study prepared and identified seven types of HBDs, and only three hydrophilic ones could form an aqueous two-phase system for targeted extraction. Among the HBDs, ChCl-Ph exhibited the lowest cooling point (−68.9 °C) and viscosity (0.014 Pa·s). Moreover, there was a higher density of hydrophilic DESs than water. For example, the densities of ZnCl_2_-Urea (1:3.5) and ZnCl_2_-acetamide (1:4) were 1.63 and 1.36 g cm^−3^, respectively [36]. Based on specific requirements, different combinations of components can be used to modify the properties of hydrophobic DESs (i.e., vapor pressure, thermal stability, liquid range, and incombustibility) [37]. The first hydrophobic DES was identified while designing water-immiscible solvents as hydrophilic DES [38]. In this work, the HDES consists of decanoic acid, which is highly hydrophobic and a quaternary ammonium salt. The alkyl chain affects the equilibrium and hydrophobicity of the DES-water system. The improvement of volatile fatty acids from diluted aqueous solutions was successfully observed by using as-synthesized DESs. Different from hydrophilic DESs, the density of hydrophobic counterparts ranged from 0.88–0.97 g cm^−3^. The hydrophobic DESs exhibited contact angles ranging from 47.9–78° and were less polar than the hydrophilic counterparts.

DESs are analogous to ILs regarding physicochemical properties, including density, electrical conductivity, miscibility, viscosity, and freezing point [39,40]. The strength of intermolecular interactions among DESs components governs viscosity, one of the most critical parameters of DESs [41]. Typically, the more polar the solvent, the more viscous it is compared to non-polar solvents. Most of the DESs exhibit relatively highly varying viscosities. It was observed that DESs with bromide anions are more viscous than chloride anions and lower viscosities DESs only contain long-chain fatty acids. The viscosity model of DESs obtained from the group contribution method has been recently reported [42]. The model predicted high accuracy of DESs viscosity using input parameters of temperature and composition and could be a powerful tool for discovering the viscosity of DESs. Regarding nanomaterial fabrication, the viscosity and surface tension of DESs is key physicochemical features that have a high impact on the interface and colloids [43]. The surface tension and viscosity are related to the cohesive forces and interactions between components of the surface [44]. Less viscosity of DESs can increase the ion species mobility and interaction forces between ions improving the formation efficiency and the nucleation of the nanostructure. Typically, the surface tension of DESs is impacted by HBD, HBA, temperature, and water content. The molar ratio of HBA and HBD of DES determines its surface tension. The high ratio increases the surface tension of DESs, investigating the effect dominating the size-dependent mechanical properties of the nanostructures [45].

The mobility of ions in DESs correlated with the fluid viscosity and conductivity, which hole theory can explain. It is assumed that there are holes in the liquid or molten state. These holes are random in size and location and undergo constant flux. The size of holes, radii of the average size void (*r*), is determined by the following equation [19]:

(1) $$4\pi \left(r^{2}\right)=\frac{3.5\;k\;T}{\gamma }$$

where k is the Boltzmann constant, T is the absolute temperature, and γ is the surface tension. Typically, the viscosity depends on the size of the voids, the size of migrating species, and the radii of cations and complexed anions. Under low temperatures, the ions transport into the vacant is reduced due to the smaller size of the holes and bigger size of the ions. It is vice versa under high-temperature conditions. The hole theory can be used to model and predict the viscosity and conductivity of DESs [46]. Most DESs are biodegradable and friendly to the environment and ecosystem [47]. Since their emergence, DESs have become a promising candidate for nanomaterial fabrication because of their economics, sustainability, dissolution ability, biocompatibility, and outstanding designability [48].

The class of natural DESs has numerous attractions as novel organic solvents. The properties of natural DESs are influenced by several factors, such as water content, temperature, and component ratio [49]. For instance, in the natural DESs containing ChCl (HBA), the viscosity in acid-based was observed to be higher than that of alcohol-based natural DESs. The alcohol-based natural DESs of glycerol and 1,2-propanediol have relatively lower viscosity. The citric acid-based natural DESs have high viscosity due to their large molecular weight due to the three carboxyl groups that could interact with HBA. The natural DESs with low molecular weight have a lower density than those with high molecular weight. It is noticed that water addition decreased DESs’ density. The possible mechanism is that the water molecules in natural DESs changed their molecular packing, decreasing density [50].

## 3. Design Strategy and Application of DES for Nanomaterial Fabrication

Since the first reported use of DESs in the green synthesis of pentacle gold nanostructure, DESs have become increasingly popular in the design strategy and fabrication of nanostructures. DESs could be an effective medium for wet-chemical processes, electrodeposition/electrochemical synthesis, and electroless deposition. DESs are involved in the process and development of nanomaterial functionalization, nano-catalytic, and nanofluid systems.

### 3.1. DES as a Solvent Medium for Wet-Chemical Approach

#### 3.1.1. Preface

The bottom-up nanomaterial fabrication is based on the principle of nucleation and growth of nanomaterials. In this approach, the atoms and molecules form nanoparticles or complex nanostructures [51,52]. The process involves assembling ions or molecules into nanoparticle formation in different theories and mechanisms [52]. Among the bottom-up methods, the wet-chemical synthesis process includes precursors, surfactants, and solvent media in the chemical reactions. Recently, the inexpensive constituents and easy preparation of DES allowed the large-scale synthesis of nanomaterials [53]. The DES-assisted wet-chemical synthesis allowed obtaining nanosize with designable specific morphology, shape, size, and properties by fine-tuning the reaction conditions [54,55]. Solvothermal/hydrothermal, template synthesis, self-assembly, and interface-mediated fabrication are the typical wet-chemical synthesis routes for nanomaterials [56]. The bottom-up chemical process involves a reaction of salts or organometallic precursors by decomposition, thermolysis, or metathesis that determine the composition of final nanostructures [57].

#### 3.1.2. Nanostructure Fabrication in DES as a Reaction Medium

DESs have been used for the fabrication of different nanostructures, including silver nanoparticles [58,59,60], gold nanoparticles [61,62,63], magnetic nanoparticles [64,65,66], Zinc nanoparticles [67], ZnSe nanoparticles [68], CZTS nanoparticles [69], upconversion nanoparticles [70,71], as well as biopolymers, such as lignin nanoparticles [72,73] and cellulose nanoparticles [74]. DESs can dissolve many different reagents and substances, including high relative concentrations of metal salts, metal oxides, and various polymers. In green chemistry, metallic nanoparticles can be biogenically synthesized using DESs to increase the efficiency of reactions [75]. DES-based methods involve novel green solvents for nanomaterial fabrication. The volatile and flammable media solvents are related to exposure toxicity during process operation and hazard explosion. DESs can also reduce the potential risk of pollution due to their negligible vapor pressure, which is safe and eco-friendly. The employment of DESs during chemical reactions can reduce the application of high temperature and pressure and decrease energy consumption for the fabrication process and harmful impact on the environment. It is noticed that although green solvents can synthesize nanostructures, some types of metal- and composite-based nanomaterials may retain their risks of accumulation in organs or production of reactive oxygen species (ROS) that directly or immediately affect human health [76]. ROS can cause biological disorders such as lipid peroxidation, DNA damage, and protein denaturation.

**Figure 2:**
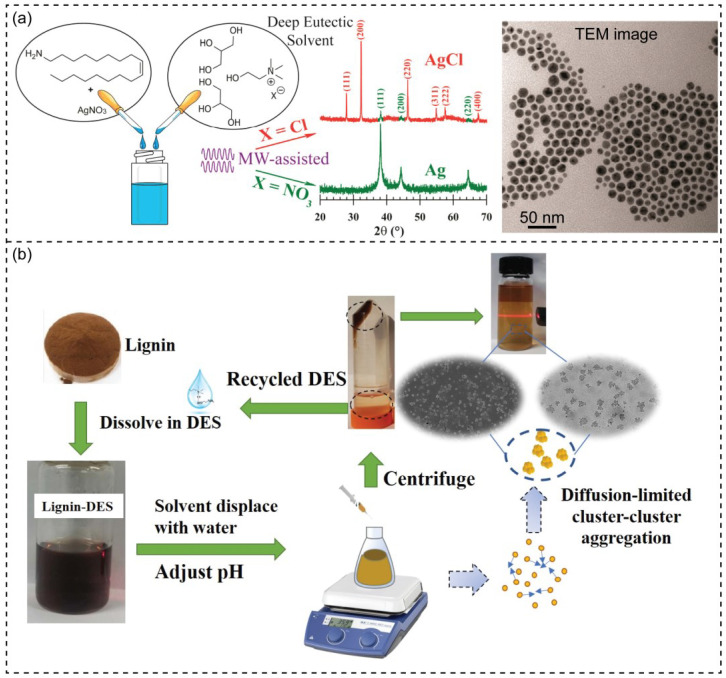
(**a**) Schematic illustration of the use of deep eutectic solvents (DESs) in fabricating organosoluble silver nanoparticles. TEM image displays the well-dispersion and high stability of as-prepared AgNPs synthesized in DES1 (choline nitrate and glycerol) after 40 days. Reprinted with permission from ref. [59]. Copyright 2018 American Chemical Society. (**b**) Design of DES as a green solvent for precipitating lignin nanoparticles with controllable size. Reprinted from ref. [72].

DES-based methods involve novel green solvents for nanomaterial fabrication. DESs control the size and shape of the nanoparticles. For instance, Dong and coworkers described the dissolution of ZnO powders and precipitation of ZnO nanostructure in a choline chloride and urea mixture [67]. A combination of facile green antisolvent and DES could on the precipitation and dimension of the ZnO nanostructure. The injection time of dissolved ZnO in DES (ZnO@DES) is a crucial factor in controlling the nanoparticle morphology. Since choline chloride and urea are more soluble in water than antisolvent, ZnO can be precipitated easily because of the significant driving force of water. The short injection time of ZnO@DES makes a competitive development of particles in a less preferred direction. Meanwhile, the provision of ZnO to support one-dimensional growth is limited in most directions by slow injection time. Xiong and his coworker reported a one-step preparation of hematite nano-spindles using DES containing choline chloride and urea [77]. In the anhydrous reaction solution, Fe^3+^ would interact with NH_3_ produced from urea in the DES medium to form Fe(NH_3_)_2_Cl_3_. The reline has been used to control the morphology and porosity of nanostructure ceria in the solvothermal method [78]. In the existence of reline, the rate of the reaction was increased. The deep eutectic-solvothermal methodology using green, economical, non-toxic solvents enabled the green synthesis of nanostructured ceria at low temperatures, resulting in the development of large-scale manufacturing. Solvothermal synthesis occurs in media solvents at wide and high ranges of temperatures and pressure producing chemical compounds from reactants [79]. DESs can be employed in various reactant systems in solvothermal methods to prepare various nanostructures. DESs can dissolve different precursors and are followed by a solvothermal process under the desired conditions. The deep eutectic-solvothermal allows for synthesizing and controlling nanostructures with less energy consumption [78]. Likewise, DESs are latent supramolecular catalysts that increase the reaction rate with solvent-driven pre-organization of the reactant as a primary mechanism of deep eutectic-solvothermal methodology. Aqueous DES was used for synthesizing magnetite (Fe_3_O_4_) nanoparticles [64]. A wet-chemical reaction was started from ferrous (II) sulfate with potassium nitrate in DES and water mixture. The morphology of nanoparticles was affected by DES concentration and the different volume ratios of water and DES. The advantages of DESs in nanosynthesis have been proven compared to the less conventional ones. Adhikari and coworkers described preparing organosoluble silver nanoparticles (AgNPs) using the free-halide DES-based wet-chemical reduction method (Figure 2a) [59]. In this work, unconventional DESs systems (i.e., choline nitrate and glycerol, choline chloride and glycerol, and choline acetate and glycerol) have been designed. The innate incompatibility of halide-based DESs was solved by nitrate-based DESs, resulting in a dispersed colloidal solution of AgNPs.

The presence of DES in nanostructure fabrication has demonstrated a role in aggregation controlling nanoparticle formation by SEM and TEM characterization. For instance, DES made of choline chloride and citric acid has been used to synthesize highly uniform and monodispersed iron oxide nanoparticles in nature [65]. The as-prepared nanoparticles are not agglomerated. In this study, DES-capped magnetite enhanced the photodegradation of organic pollutants and inhibited excellent anticancer behavior. Currently, DES composed of choline chloride/glycerol (ChCl/Gly) showed an effect on the dispersion of carbon nanotubes in water [80]. The aqueous DES system disaggregates carbon nanotubes into smaller bundles that assist in controlling CO_2_ absorption. Superior dispersion of single-wall carbon nanotubes (SWNTs) in water was provided by the net negative charge surface of as-prepared DESs via electrostatic repulsion of sonication. The optimized dispersions were explored through the study of ChCl/Gly concentrations (% mass in water), SWNTs concentrations (mg/L), and sonication energy (J/mL). Moreover, the interdisciplinary application of DESs has been proven in the self-assembly of surfactants. The phenomenon involves the micelle formation of cationic surfactants in designed DESs. The DES-surfactant behaviors provide potential application in the fields of material synthesis due to their impact on the degree of ionization, interface, and thermodynamic parameters [81].

DESs play a role in nanoparticle stability and well dispersity. The reline containing choline chloride, and urea enables the effective formation of highly crystalline and nanosized fluorapatite nanoparticles [82]. The ionic strength and 3D-bulky alignment of reline can control the growth and electrostatic stabilization of as-synthesized fluorapatite nanoparticles. Notably, the electrostatic stabilization was accelerated by the high ionic strength of DESs, blocking or weakening inter-particle collisions. Meanwhile, the steric repulsion coordination between particles’ surfaces resulted from the bulky geometry of DESs, which decreased agglomeration, leading to enhanced mono-dispersion of particles [82,83,84].

In solvothermal methods, DESs act as solvents and catalysts [85]. Fast synthesis of ceria nanoparticles (CeO_2_ NPs) has been successfully achieved using reline as a chemical process solvent [86]. The mixture of choline chloride and urea (1:2 molar ratio) was used to make reline. The reaction medium’s molecular structure boosted the cerium oxycarbonate’s fast nucleation growth. The quick formation of CeO_2_ NPs was achieved in reline without the addition of corrosive NaOH or a large amount of input energy. In this study, a varying amount of water was added to DESs to control CeO_2_ morphology. In this case, the mixture of DESs and water works as greener processing and reaction media where the reaction rapidly occurs under reasonable conditions. It has been reported that the suitable addition of water can modify and improve the physicochemical properties of DESs, such as viscosity. However, a certain additional amount of water in DESs should be carefully studied due to the alteration of DES’s structure even at low hydration levels [87]. Although the water molecules show high interaction with different DESs components through hydrogen bonding, the properties of DESs can be maintained to reasonably large water content [88]. This situation controls and enhances DES properties and features while maintaining their functions [89,90]. In hydrothermal synthesis, the existence of urea in DES also triggered the formation of Ce(OH)CO_3_ and Ce_2_O(CO_3_)_2_, which produced CeO_2_ [91,92]. Four kinds of ternary DESs (T-DESs) have been reported as green solvent mediums for fabricating silver nanoparticles [60]. These T-DESs were prepared by evaporating a mixture of zin chloride with HBDs (glucose and fructose) and amino acids. T-DESs work as a reaction medium to obtain AgNPs by chemical reduction method. The presence of urea may influence the chemical reaction by enhancing the oriented attached mechanism [89,90].

DESs have the capacity dissolution of different biopolymers, including cellulose, lignin, starch, and chitin. Recently, highly uniform lignin NPs were prepared by a simple and environmental method (Figure 2b) [72]. Lignin is one of the most abundant biopolymer that has limited solubility. In this work, the DESs (choline chloride and ethanolamine/ethylene glycol /lactic acid) were used as “green solvents” with the ability to dissolve 20–40% industrial lignin and the formation of its nanoparticles. DESs and water are miscible, and an interface membrane at two-phase between DESs and water can be formed by highly hydrophobic lignin molecules in the soluble lignin-DESs [72,93]. In this case, DESs function as less hydrophobic surfactant dispersed in colloidal suspensions. In another study, the natural acidic DES comprising choline chloride and oxalic acid dihydrate is used to obtain cellulose nanocrystals, of which the crystallinity yields 43.6 ± 1.9% [74]. The applications of hydrogen bond donors and acceptors in different ratios can prepare acid DESs that hydrolyze the amorphous parts of cellulose. Recently, Lai and coworkers used cellulose nanocrystals (CNCs) and DES 3D printable ionogels [94]. The protocol resulted in strong physical and printable inks by reducing the concentration and inducing the desulfation of CNC at high temperatures. The lifetime of ionotropic was also increased because of high mechanical toughness and self-healing capability.

### 3.2. Nanosized Reticular Material Fabrication

Recently, unique structures and properties of reticular materials have attracted considerable interest in catalysis, energy storage, and sensors [95,96]. The flexibility of composition, structure, and pores of metal-organic frameworks (MOFs) and covalent organic frameworks (COFs) are typically associated with and determined by the fabrication method and design strategy [97]. The presence of DESs in the fabrication of reticular nanomaterials can control the formation of MOFs in many ways. DESs could act as solvents and structure-directing agents or template-delivery agents for the architecture of MOFs [98]. In some cases, DESs components could be involved in the structure formation, and their decomposition products formed under ionothermal conditions.

#### 3.2.1. MOF Fabrication

DESs can improve the chemistry of MOFs. The components (i.e., cations, anions, and neutral ligands) of DES have involved the structure formulation of MOFs [99]. They are involved in the self-assembly process exclusively or in combination with other components to form new types of chemistry. Notably, structure-directing effects can be exerted by neutral ligand molecules (i.e., urea). For instance, they can bind to metal sites, and removing neutral ligands could form porosity and open metal structures. Zhang and coworkers have described a series of MOFs synthesized using three DESs. For example, different distinct framework topologies can be generated using the self-assembly of lanthanide ions, 1,4-benzene dicarboxylate, and components of DESs [99]. The reported mechanisms include the integration of cations-anions/neutral ligands. The new role of DES in MOF fabrication has also been presented in the framework of zinc(II)-boron(III)-imidazolate porous (ZBIF1) with an unusually pentagonal structure [100]. Either action (choline) or neutral ligand (m-urea) is integrated into ZBIF1. The Zn-Cl bonding in ZBIF1 is typically inaccessible in conventional hydrothermal synthesis but can be achieved in DES. This can be considered the first metal-imidazolate framework fabricated from DES.

In aluminophosphate synthesis, the presence of DESs allows controlling the mineralized concentrations to be retained and work as a template for delivering the reaction mixture [101]. The choline chloride/urea DES (melting at 12 °C) has been employed to create a zeotype framework (SIZ-2) [102]. The template configuration and the charge balance of the framework were supported by ammonium resulting from decomposition with urea of DES. The maintenance of Al-O-P alternation was observed. However, the chemical formula indicated that SIZ-2 is an interrupted structure, which has the benefit of comprising anionic frameworks. Recently, transition MOFs based on oxalate-sulfate anions in DES have been described by Xiong and coworkers [103]. A mixture of chloride and oxalic acid with a molar ratio of 3:7 was used as the reaction medium to make a series of iron oxalatosufate networks. Oxalate and sulfate anions were employed in the structure as connecting ligands which performed the role of DES to act as an organic linker in the construction.

A new porous and bioinspired structure of Zr-based MOF, called UiO-66-Urea, was formed following a post-synthetic coating and hydrogen bonds between UiO-66-Urea and choline chloride [104]. Various HDBs can determine the different MOF frameworks in DES [105]. The assembly of metal ions and conjugating ligands is significantly affected by HBDs. This work created seven new transition MOFs from three DES mixture compounds of choline chloride and urea/e-urea/m-urea. HBDs have been proven to work in multiple roles in formatting various nanostructures, such as urea and e-urea acting as reactants by bonding to the metal ions. The generation of NH_4_^+^ and CH_3_NH_3_^+^ cations through the hydrolysis of urea and m-urea contributes to the construction of the frameworks and connection between metal ions and ligands as the templates or charge-balancing agents.

#### 3.2.2. COF Fabrication

**Figure 3:**
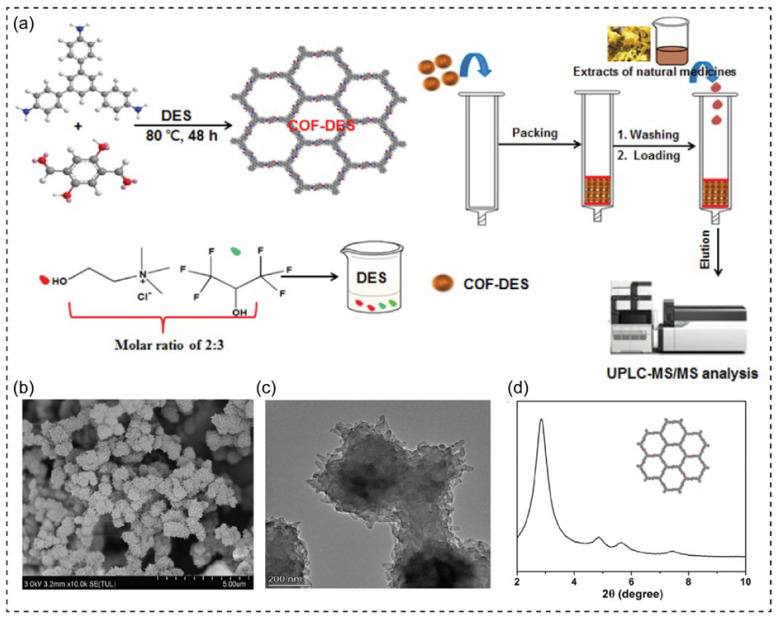
(**a**) The design and application of deep eutectic solvents (DESs) as a reaction solvent for fabricating high crystallinity COF-DES. (**b**) SEM image, (**c**) TEM image, and (**d**) PXRD pattern of as-prepared COF-DES. Reprinted with permission from ref. [108]. Copyright 2021 American Chemical Society.

The assembly of organic components is one of the major principles for fabricating COFs [102]. The strong covalent bonds in 2D and 3D crystal porous materials assist COFs formation [106]. DES contributes to the crystallinity of COFs. For example, DESs have been used as green media to avoid high temperatures, hazardous organic solvents, and long reaction times in fabricating crystalline 2D and 3D COFs [107]. This study used three DES-based ChCl (ChCl/Glycerine, ChCl/Urea, and ChCl/Ethylene glycol) to enhance the crystallinity at an optimized concentration. The DESs were designed to control the structures and properties by changing HBD and HBA. The tetrabutylammonium bromide and imidazole-based DESs were recently used to fabricate carboxyl-functionalized COF (TpPa-COOH) for dye absorption [106]. The as-prepared TpPa-COOH had open channels and exhibited good porosity and crystallinity under the low vapor pressure of DESs. Recently, Gao and coworkers described COF-DES inferred from 1,3,5-tris(4-aminophenyl)benzene and 2,5-dihydroxyterephthalaldehyde (Figure 3) [108]. The DES has been used as a reaction medium instead of organic solvents to adjust pores and specific regions with high crystallinity. The interferences of some compounds can be prevented by the COF-DES combination, which is used for the selective adsorption of flavonoids.

### 3.3. Electrodeposition/Electrochemical Synthesis

#### 3.3.1. Principles and Characterization

Electrodeposition is a convenient, flexible, low-cost, and environmentally safe method for fabricating 2D and 3D materials of nanostructure coating and films [109,110,111,112]. The electrodeposition process can fabricate different nanostructures and thin film coating using a simple experimental setup and operation. It can avoid using aggressive chemical-reducing agents [110]. DESs have broad applications for electrodeposition and electrochemical fabrication of polymers, metals, alloys, and semiconductors in electrochemistry. Electrodeposition is a versatile method for fabricating nanomaterials to improve materials’ characteristics and decorative and functional properties [55]. It has been observed that the electrodeposition process is challenging to achieve in an aqueous solution due to water hydrolysis, which can cause a hydrogen evolution reaction and low electrodeposition current efficiency [113]. The electrodeposition in aqueous solutions is a negative reduction potential [114], while DES provides innovative potential and relatively high conductivity for green and effective electrodeposition for nanostructure architecture [115]. The nanostructure of materials can be characterized by morphology, chemical composition, and electrochemical analysis. Other physical and chemical properties have been considered for studying properties, such as magnetism, resistance, thickness, and corrosion. Typically, the surface morphology and chemical composition of the nanostructures is observed by scanning electron microscope (SEM), transition electron microscope (TEM), and atomic force microscopy (AFM) [112,116,117]. The energy dispersive X-ray spectrum (EDS), X-ray diffraction (XRD), and X-ray photoelectron spectroscopy (XPS) are used to identify the structure and composition of the materials [118,119,120]. The electrochemical behavior of the material can be obtained and analyzed by several parameters (i.e., cyclic voltammetry, electrochemical impedance spectroscopy, chronopotentiometry, and potentiodynamic polarization study) [118,121,122].

#### 3.3.2. Electropolymerization

Electrochemical methods are used to design polymer structures by adjusting the electrochemical parameters [123]. The generated polymer may be directly deposited on an electrode surface in its doped or undoped state under DESs media conditions. Hosu and coworkers described the electrodeposition of nanostructured phenazine polymer poly(methylene blue) (PMB) from ethaline DES on glassy carbon electrodes [124] and on carbon nanotubes [125]. The polymer was obtained in different DES media at different scan rates (ranging from 50–500 mV/s) to find the optimized conditions for nanostructured PMB film formation [124]. The DES and scan rate influenced the formation of spherical polymer nanostructures. The nanostructures were not well-defined for the 50 mV/1 formation scan rate, and the diameter ranged from 160–300 nm. For 150 mV/1, ordered nanostructures were obtained with sizes ranging from 40–70 nm and granular films. For 500 mV/1, some cauliflower growths were obtained, and spherical nanostructures’ diameter ranged from 50–160 nm. The scan rate was a crucial factor influencing the nanostructure polymer film’s structure, morphology, and electrochemical properties.

Different choline-based DESs have been studied for the electrosynthesis of polymers, such as polyaniline (PANI) [126]. Under ethaline DES conditions, highly crosslinked PANI nanoribbons were developed with a dimension of 80 nm and pores of around 150 nm. The structure of PANI-Gly was more nanoparticulate with a pore size of 100–250 nm. No significant polymerization of aniline can be claimed for PANI-Rel after twenty-four scans. The electropolymerization of PANI was studied on propeline DES containing a molar ratio of 1:2 of choline chloride and 1,2-ethanediol [127]. It was hypothesized that DES brings polymers with an electrochemical and optical performance at much lower economic and environmental costs. The polymer films were obtained from highly branched and crosslinked structures of PANI nanoribbons to show the correlation between DES and polymer performance, altering the optical and electrical properties. The electropolymerization of poly(3,4-ethylene dioxythiophene) (PEDOT) was obtained in DESs that contain a reline of choline chloride–urea and an ethaline of choline chloride–ethylene glycol [127]. The electrodeposition of PEDOT also was reported in DESs with perchloric acid [128]. PEDOT fabricated in DES medium comprised of choline chloride, urea (reline), and HClO_4_ showed the best performance for detecting ascorbic acid, dopamine, and uric acid.

Recently, the growth of PANI in oxaline DES inferred from cyclic voltammetry, and electrochemical quartz crystal microbalance was reported [129]. The cycling of the voltage (ranging from 0.1–1.2 V) and 0.25 M aniline in Oxaline-based DES were used to deposit PANI. In DESs, the insertion of anions increases the oxidation of the mass of the polymer film. Remarkably, the mass increased on a cathodic sweep.

#### 3.3.3. Electrodeposition of Metal-Based Materials

DESs in the electrodeposition and electrochemical process can be applied for different purposes, such as functional surfaces, to achieve required hardness, brightness, magnetism, electrocatalysis, corrosion, and wear resistance [130,131]. The electrochemical coating deposited on the surface could improve substrate materials’ solderability, lubricant properties, and electrical conductance [132]. Because of the passivation of the electrode, it is challenging to fabricate many metals and their alloys from the aqueous solvents, but it is easily electrodeposited in DES. Several metal nanostructures based on nickel, copper, zinc, aluminum, and their alloys fabricated in DES have been reported [55,115].

**Figure 4:**
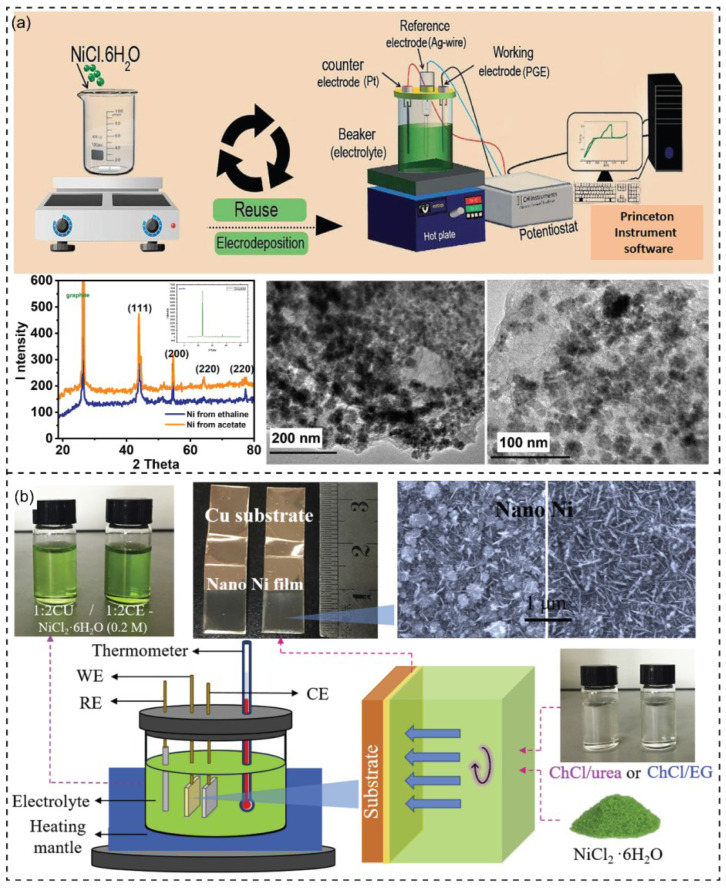
(**a**) Scheme of the electrodeposition of NiNPs in ethaline. XRD pattern of synthesized Ni/PGE in DES (ethaline) and in acetate bath. The inset graph displays the XRD pattern of PGE. TEM images show the deposited NiNPs from DES. Reprinted from ref. [134]. (**b**) Scheme of the DES-assisted electrodeposition of nanostructure Ni films on Cu substrate. The inset images show the electrolytes and SEM of as-fabricated nano-Ni films. Reprinted with permission from ref. [136]. Copyright 2018 Elsevier.

The electrodeposition of various metals using DESs is an attractive topic due to their cost-effectiveness, easy preparation, and biodegradability [133]. Table 1 shows the common use of DESs containing choline chloride for electrodepositing nanostructures of metals and alloys. For example, performing electrodeposition of Ni from DES is more effective than from an aqueous solvent [134]. The electrodeposition of Ni in DESs generally does not require a surfactant to obtain a nanocrystalline structure with low surface roughness. It has been reported that DESs provided a deposit site of nickel to different crystal structures [115]. Elsharkawaya and coworkers have reported the electrodeposition of Ni nanoparticles from DESs with a size of 20 nm and uniform needle flower structure (Figure 4a) [134]. The deposition process in ethaline required an overpotential of −154 mV and 350 mV for the hydrogen evolution and oxygen evolution reaction, respectively. In another study by Gu and coworkers [135], the nanostructured Ni films were synthesized by electrodeposition with adjusting constant, pulse, and reverse pulse voltage to create micro/nano binary surface of nanosheets, aligned nanostrips, and hierarchical flowers. The structure and chemical composition of the metal film’s surface exhibited superhydrophobicity spontaneously without modifications. The study found that the process temperature was essential for the surface roughness and topography of the Ni films. Specifically, at 90 °C, the Ni nanosheet thickness and the size of Ni grains were 10–20 nm and 10–50 nm, respectively. The study revealed that less viscosity of DES could increase ion species mobility, enhancing Ni’s deposition efficiency and nucleation.

**Figure 5:**
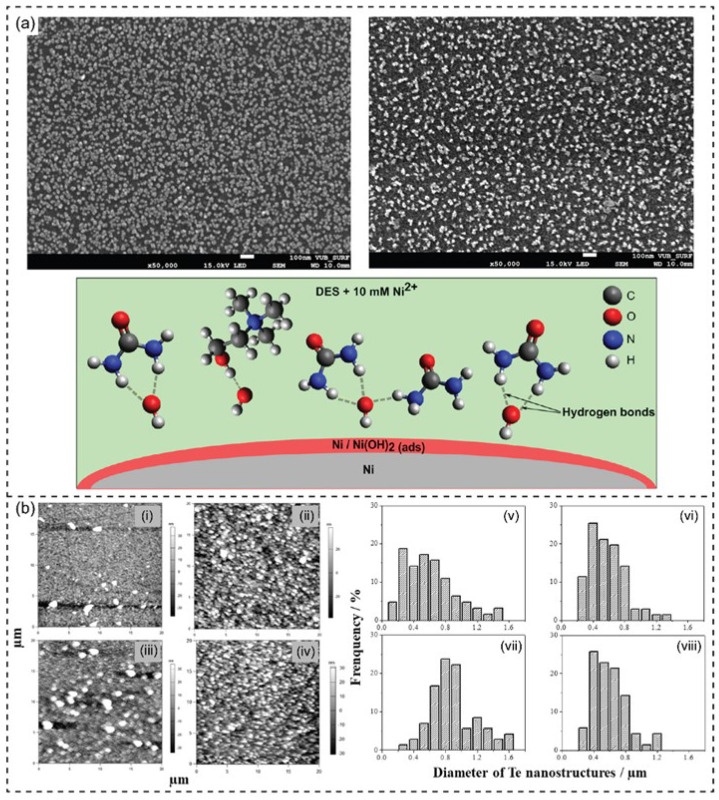
(**a**) FE-SEM images of the as-prepared nickel nanostructures by electrodeposition process from DES (ChCl-U) on glassy carbon (**above**) and schematic of the formation of hydrogen bonds between hydroxides and DES components-based ChCl-U (**below**). Reprinted with permission from ref. [140]. Copyright 2017 American Chemical Society. (**b**) AFM images of Te electrodeposits on Au-coated FTO electrode from DES of ChCl-EG at (i) 30 °C (E = −0.04 V for 1.20 s) and (ii) 80 °C (E = 0.16 V for 0.50 s); and DES of ChCl- U DES at (iii) 30 °C (E = −0.28 V for 0.80 s) and (iv) 60 °C (E = −0.22 V for 0.50 s). Histogram displays the diameter distribution of 1D Te nanostructure obtained from ChCl-EG at (v) 30 °C and (vi) 80 °C; and from ChCl-U at (vii) 30 °C and (viii) 60 °C. Reprinted with permission from ref. [141]. Copyright 2019 Elsevier.

DESs demonstrate excellent properties of electrolyte and electrochemical behaviors. DES can be a potential electrolyte that plays a critical role in metal and metal nanoparticle electrodeposition. For instance, Ni and Sn nanoparticles were synthesized using a different type of DESs as a synthetic medium and the electrolyte of the electrochemical fabrication method [137]. Wang and coworkers have described the electrodeposition of nano-Ni films using DES (Figure 4b) [136]. Similarly, Gu and coworkers have reported developing nanocrystalline Ni by electrodeposition in choline chloride and ethylene glycol [138]. Li and coworkers have reported the electro-codeposition of Ni-SiO_2_ nanocomposite coating by DES-ChCl-EG to improve the corrosion resistance [139]. Co-deposition of SiO_2_ into the metal pattern from an aqueous solution is challenging. However, the study revealed the stability of SiO_2_ nanoparticles in ChCl-EG without additives. Moreover, the existence of SiO_2_ nanoparticles significantly affected the nucleation mechanisms of Ni. Consequently, the fabrication of metal matrix nanocomposite coatings can be processed effectively using DES. The Ni nanostructure has been deposited on the carbon substrate from DES of choline chloride and urea (Figure 5a) [140]. It was observed that hydrogen bonds were formed between hydroxide groups and the DES components. Additionally, a dense distribution of Ni nanostructures could be observed.

The electrodeposition of 1D tellurium (Te) nanostructures on the gold surface have been described in DESs based on ChCl-U and ChCl-EG [141]. The electrocrystallization followed a 3D-bulk coagulation mechanism with growth control by diffusion. The plating solution of ChCl-EG and ChCl-U exhibited a sweeping potential between 0.6–1.0 V of cyclic voltammograms. The Te nuclei and the nanostructure growth were gradually formed on the substrate in DES mediums (Figure 5b). The surface morphology of Te electrodeposited films was observed with a uniform distribution of hexagonal Te rods on the Au surface. In other work, Hammons and coworkers have described using DES for the electrodeposition of Pd nanoparticles into 2D superstructures [142]. The DES was prepared by recrystallizing choline chloride and urea (ratio of 2:1). Two temperatures were used for the electrodeposition process due to their influence on the viscosity and conductivity of the DES. The SEM characterized the nanoparticle morphology to show large particles of ~20 nm at 44.5 °C and small particles of ~10 nm at 32.5 °C. The role of DES was confirmed as a stability agent of the nanoparticles in the electrochemical process. Similarly, direct and pulse current electrodeposition of Pd from a mixture of DES component and palladium (II) chloride was reported [143].

The role of DES in triacontahedral Pd nanocrystals (C-DTH Pd NCs) was demonstrated [144]. The DES took part in the growth of C-DTH Pd NCs by adjusting the square wave potential’s upper limit. In principle, DESs were excellent soluble solvents for metal and metal oxides. It was reported that the metal oxides usually are insoluble in common room-temperature ionic liquids but have high selective dissolution in DES [145]. The electrocrystallization behavior of copper in DES was studied previously [146]. The copper (I) oxide was introduced into the urea melt by dissolving Cu_2_O. The DES performed potential for inexpensive electroplating of copper warrants a copper salt reaction. In another study, the preparation of copper nanoparticles (CuNPs) in DES was described [147]. The cyclic voltammogram analysis was performed to analyze the electroreduction of copper in DES with a coefficient of 0.78 × 10^−8^ cm^2^ s^−1^. It was found that depending on the voltage, the CuNP sizes were increased from 28 ± 7 and 57 ± 6 nm at 2.5 and 2.2 V, respectively. The ChCl-urea-based DES exhibited a potential replacement of the traditional hydrogen evolution for fabricating CuNPs. Vertically aligned carbon nanotubes having nano-chromium magnetic domains were developed using DES [148]. Using chromic acid electroplating for electrodepositing electronegative metals from an aqueous solution is sufficient. In a eutectic mixture of ChCl and CrCl_3_.6H_2_O, chromium’s conformable electroplate nanomagnetic domains onto carbon nanotube were achieved. Cvetkovic and coworkers have shown that using DES to control the electrodeposition of aluminum (Al) with Al grains varied in size from nano- to micro-meters [149]. The usage of DES-based ChCl-U for the electrodeposition of aluminum was addressed [150,151]. The nucleation and growth of Al were studied in ChCl-U [151]. In another work, gold and silver were recycled from anodic slime using choline chloride and ethylene glycol (molar ratio of 1:2) [152]. The grain silver and gold crystals were ~28.04 and 10.5 nm, respectively. Sides and coworkers have presented nanoscale manganese (Mn) thin films deposited on disk electrodes from a mixture of ChCl and U [153]. The deposited metallic Mn films were observed with a minimum thickness of 400 nm and were highly conductive. Mn deposition could be resumed at low current efficiency [117]. Manuel and coworkers have reported the electrodeposition of FeNPs onto the electrode surface of HOPG from Fe(III) ions in ChCl-U DES [154]. The electrodeposition of pure metal and electrochemical synthesis of nanostructure on the surface of electrodes using DESs has been considered an innovative methodology to control better the size, morphology, and surface chemical composition of the nanostructure materials.

**Table 1:** Electrodeposition of nanostructures of metals and alloys with DESs-based choline chloride.

Metal or Alloy	Type	DES Component	Molar Ratio	References
Ni	Nanoparticle	ChCl-EG	1:3	[134]
Ni	Nanosheet	ChCl-EG	1:2	[135]
Ni	Nanostructure and nanofilm	ChCl-U ChCl-EG	1:2 1:2	[136,138,140]
Te	Nanoparticle	ChCl-U ChCl-EG	1:2 1:2	[141]
Pd	Nanoparticle	ChCl-U	1:2	[142]
Pd	Nanocrystal	ChCl-U	1:2	[143,144]
Fe	Nanoparticle	ChCl-U	1:2	[154]
Cu	Nanoparticle	ChCl-U	1:2	[147]
Au-Ag	Nanoparticle	ChCl-EG	1:2	[152]
Mn	Nanofilm	ChCl-U	1:2	[153]
Fe-Cr	Nanocrystal	ChCl-EG	1:3	[155,156]
Cu-Au	Bimetallic nanostructure	ChCl-U	1:2	[157]
Ni-Mo	Nanocrystal	ChCl-PG	1:2	[158]
Au-Pt	Nanoflower	ChCl-EG	1:2	[159]
Ni-Co	Nanofilm	ChCl-EG	1:2	[160]
Pd-Co	Nanoparticle	ChCl-U	1:2	[161]
Co-Pt	Nanocrystal	ChCl-U	1:2	[162]
Ni-Co-Sn	Nanocrystal	ChCl-EG	1:2	[163]

ChCl: choline chloride, EG: ethylene glycol, U: urea, PG: propylene glycol.

#### 3.3.4. Electrodeposition of Alloys

**Figure 6:**
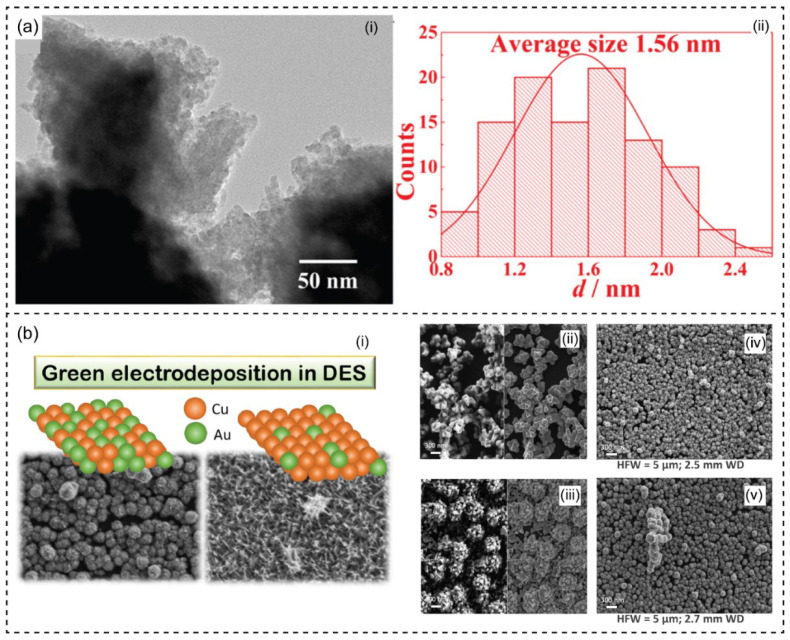
(**a**) TEM image (i) and size distribution histogram (ii) of Fe-Cr alloy fabricated in ChCl-EG DES. Reprinted with permission from ref. [155]. Copyright 2022 Elsevier. (**b**) Preparation of Cu-Au nanostructures by co-electrodeposition in DES. Green electrodeposition of Cu and Au high surface area on glassy carbon (GC) in DES (i); FE-SEM images of Au deposit (ii) and Cu deposit (iii); Cu-Au co-deposit in stationary conditions (iv), and stirring at 200 rpm (v). Reprinted from ref. [169].

The electrodeposition and electrochemical behavior of alloys in DESs have been extensively addressed, such as Fe-Cr [155,156], Cu-Au [157], Ni-Mo [158], Au-Pt [159], Ni-Co [160], Cu-Sn [164], Zn-Sn [165], Pd-Co [161], and Co-Pt [162]. The electrodeposition of more than one metal often forms different structures and properties [166,167]. The electrochemical behavior and process are similar to pure metal deposition. The physical and chemical character parameters differ with respect to the temperature, composition, potential, current density, and plating bath. It has been reported that the ultimate aprotic liquids (e.g., DESs) for the electrodeposition of alloys can avoid the hydrogen evolution issue that limits the potential application of an aqueous solution [55,140]. Recently, the nanoscale Fe-Cr alloy was achieved by electrodeposition in ChCl-EG without adding any additives (Figure 6a) [155,156]. CrCl_3_.6H_2_O and FeCl_2_.4H_2_O were dissolved in ChCl-EG DES. The nucleation of Fe, Cr, and Fe-Cr alloy was more instantaneous. However, the nucleation of Fe or Cr was less efficient than the Fe-Cr combination, demonstrating the co-deposition in DES. Notably, a larger concentration ratio of Fe(II) and Cr(III) (i.e., ratios of 1:5–3:3) exhibited high electrodeposition of the nanocrystal alloy. The scratched as-prepared Fe-Cr alloys comprised a dense surface with a mean diameter of 1.56 nm. The reaction temperature has significantly impacted the electrical conductivity and viscosity of DESs which has been discussed above to influence the mobility of ions and the interaction force between ions [168].

Fabrication of bimetallic nanostructured Cu-Au by co-electrodeposition in DES has been published as a green synthesis approach (Figure 6b) [169]. Tunable nanostructure and composition were carried out in green ChCl and U to tailor features of the deposits (i.e., size, morphology, and elemental composition). Different bath compositions were prepared in the DES solution. The formation of the nanostructure of single Cu and Au and bimetallic Cu-Au electrodes were successfully obtained in the ChCl-U. Typically, the broader potential of DESs in electrochemical applications relates to their negligible hydrogen evolution and thermal stability on the electrode. Niciejewska and coworkers have described a homogenous nanocrystalline structure of Ni-Mo electrocoating in DES-based ChCl-PG [158]. The use of DES can overcome the limit of water-based galvanic baths regarding the significant release of hydrogen from the water bath. It was also reported that the deposited nanocrystals in DES exhibited low surface roughness compared to aqueous solutions and sufficient corrosion resistance [170]. In another work, Li and coworkers have presented an electrochemical fabrication of AuPt nanoflowers in DES-based ChCl-EG [159]. The one-step electrochemical reduction was carried out to control and form bimetallic AuPt alloy nanoflowers. The role of DESs was indicated as solvents and shape-directing agents. DESs are considered green solvents that have been widely applied in the reaction process of nanoparticle fabrication. Moreover, the electrodeposition of Ni-Co alloys was successfully conducted in ChCl-EG DES at room temperature [160]. The Ni-Co alloys had a cubic structure and refined grains. The grains of deposits were identical and had a size of about 20 nm. Recently, the fabrication of Pd-Co alloy nanoparticles (PdCoNPs) has been described to obtain by an electrochemical process in DES-based ChCl-U [161]. The potentiostat investigated the formation of PdCoNPs on glassy carbon (GC) electrodes from their precursors dissolved in the ChC-U. The obtained PdCoNPs had an average size of 30 ± 4 nm and particle number density of (4.23 ± 0.33) × 10^10^ PdCoNPs cm^−2^. The electrodeposition of Ni-Co-Sn alloy was investigated from ChCl-EG DES to obtain a crystallite size of 7–21 nm [163]. By ultimate DESs, alloys’ electrodeposition and the electrochemical process can be controlled to obtain the nanostructured materials without requiring additives and stabilizing agents. The materials’ properties depend on the experimental setup, including plating bath composition, current density, potential, and temperature. Several metals and alloys have been prepared in DESs to avoid hydrogen evolution reactions.

### 3.4. Electroless Decomposition

Electroless decomposition is based on autocatalysis [171]. Electroless deposition is a chemical process that depends on the oxidation of chemical compounds and the reduction of metallic ions [172]. The redox in DESs for electroless deposition is a versatile technique for metal plating and metal-based material development [173]. Electroless deposition methodology can be classified into three categories: galvanic displacement reaction, reducing agent usage, and disproportionation reaction. Electroless plating baths typically contain metal ions resource, reducing agents, stabilizers, and buffering agents. In galvanic displacement, the base materials are dissolved in the solvent, and the metallic ions are usually reduced without requiring reducing agents [174]. For instance, Wang and coworker have described dipping Au deposition from choline chloride and chloroauric acid (ChCl-HauCl_4_) [175]. In DES, the ChCl was used as a ligand, and HauCl_4_ was the source of Au. The study revealed a decrease in deposition rate by an increase in ChCl concentration due to its effect on the viscosity and formation complex with Au(III). The lower ChCl concentration allowed a shift in equilibrium because the increase of free Au induced the collision between Ni and Au. In optimized conditions, SEM and XRD obtained the Au coating layer with a thickness of about 50 nm in DES.

Electroless deposition in DESs has been applied for various surface coatings, such as pure metals [176,177,178] and alloys [179,180]. Recently, the electroless deposition of Rhenium from reline DES-based ChCl-U has been demonstrated [176]. The evaluation of corrosion performance was tested by voltammetry and chronoamperometry. Similarly, Ni’s electroless and electrolytic deposit in DES-based ChCl-EG and ChCl-U has been reported [181]. The cathodic current efficiency of Ni deposition was 97%. Yang and coworkers have presented N-decorated h-BN powders using ChCl-EG through electroless plating [182]. It was observed that an increase in Ni plating time and the average size of spheroid Ni particles ranging from 10–1000 nm caused a high deposition of Ni particles on the h-BN surface. In another work, electroless Ni-P coating was utilized in DES-based ChCl-EG in wide-range temperatures to improve the properties of Al alloy in terms of corrosion resistance and mechanical features [179]. ChCl-EG was used as deposition media for the Cu dipping layer on Ai alloy with a hierarchical formation and electroless Ni-P process. The globular protrusion of the deposited layer was rough and explored numerous nanosized particles. The potential of ChCl-EG was reported to facilitate sustained galvanic alternative deposition with high consistency, which is challenging to achieve in an aqueous reaction [177]. The design of immersion plating from DESs relies on the potential galvanizing alteration between the ions and the substrate. The reduction of metal base material in DES occurred with or without reducing agents. The electroless process by simple immersion of the substrate in DESs contains metal ions base materials, and homogenous film can be deposited fast [19]. In some cases, a neutral plating bath triggered by DESs could minimize the likelihood of corrosion occurring and reduce the possible formation of black-pad caused by excessive corrosion.

### 3.5. Nanostructure Functionalization

**Figure 7:**
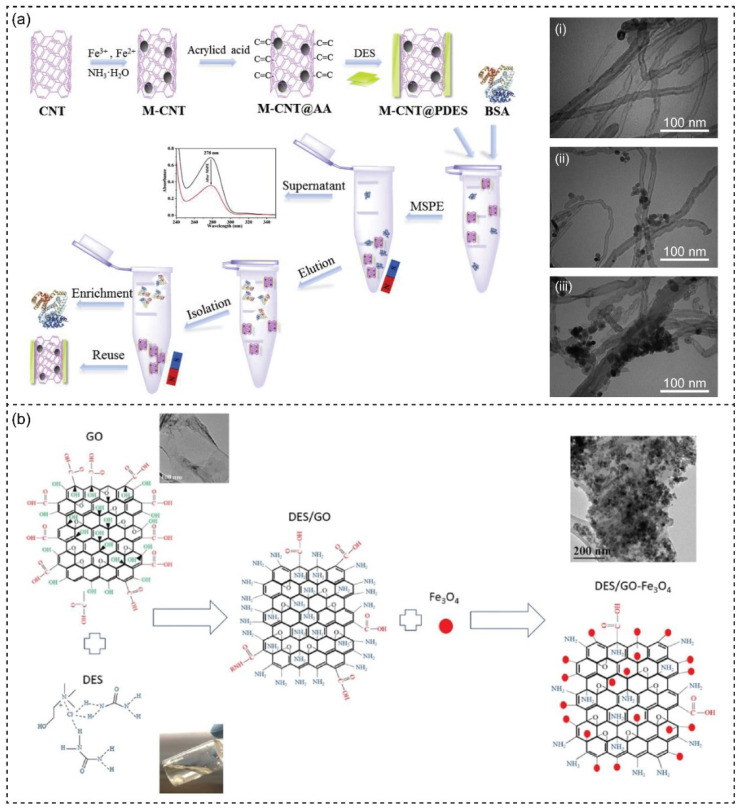
(**a**) Schematic illustration of the fabrication process of magnetic carbon nanotubes modified with polymeric DES (M-CNT@PDES) and its design application for extraction of bovine serum albumin (BSA). TEM images of CNT (i), M-CNT (ii), and M-CNT@PDES (iii). Reprinted with permission from ref. [194]. Copyright 2020 Elsevier. (**b**) Schematic illustration of the conjugation of magnetic nanoparticles (MNPs) onto graphene oxide (GO) using ChCl-U and its application for lead(II) and methylene blue removal. Inset TEM image of bare GO nanosheets and DES functionalized GO nanosheets (DES@GO). Reprinted with permission from ref. [199]. Copyright 2020 Elsevier.

The process of adding new functional groups to the surface of the materials via chemical or physical interaction is called functionalization [183,184]. DESs have been recognized as functionalization agents for nanomaterial engineering. Due to the various combinations and molar ratios of HBA and HBD of DES, different interaction types, such as weak, non-covalent, anion exchange, and π-π/hydrogen bonding takes place to contribute to the properties of functional medium [185,186]. This phenomenon could create the unique interaction of DES and target compound, making it a potential application in synthetic chemistry and material functionalization [187]. In polymer-based materials, DESs can be used as a functional additive that acts as a template and/or ligand supplier [55,188]. Several ChCl-based DESs have been designed to be used as plasticizers for producing polymers [189,190,191,192]. DESs functionalized polymers have been reported for easy and efficient biocatalysis [193]. The poly(vinyl pyrrolidone DES)s were obtained to determine the best L-asparaginase absorption. In another work, Ni and coworkers have described magnetic carbon nanotube (M-CNT) modified polymeric DES (PDES) for solid-stage extraction of bovine serum albumin (Figure 7a) [194]. Acrylic acid was used to modify M-CNT, resulting in M-CNT@AA coated with the designed DES by free radical copolymerization. After the functionalization of the DES polymer layer, a reduction from 52.89 to 43.72 emu/g of the magnetic strength of the magnetic nanoparticle was observed.

More reports have described the functionalization of CNT using DESs [195,196,197,198]. Alomar and coworkers studied DESs composed of ChCl and six different HBDs to use as functionalization agents on CNT for heavy metals absorption [195]. DESs have been successfully applied for multi-wall CNT (MWCNT) functionalization with significantly improved MWCNT structure and chemical properties. Several different designed DESs were prepared and studied. To study electrochemical sensing, the ChCl-U functionalized MWCNT was applied [196]. Similarly, DES, comprised of allyl triphenyl phosphonium bromide and glycerol, has been prepared to modify CNT as a novel absorbent of mercury from water [197]. The DES functionalized nanostructure significantly enhanced their stability and good dispersion in the target solvents. The effects of DESs on the surface of CNT were intensively investigated [198]. In this work, novel absorbents were created from DESs and CNT to remove arsenic ions (As^3+^). Specifically, active sites on the CNT surface and adsorption of As^3+^ were enhanced in the presence of DESs.

Applying DESs for graphene oxide functionalization and nanoparticle conjugation has recently been investigated and applied in drug delivery, water treatment, catalysis, and biosensor application [199,200]. The modification and addition of functional groups to the surface of graphene were investigated with eighteen different types of ammonium- and phosphonium-salt-based DESs [200]. The study indicated the change in the surface chemistry of materials after DES functionalization according to the new hydrophilic functional groups and different reducing abilities of the used DESs. In another work, DES-based ChCl and U have been used to control the Fe_3_O_4_ conjugate in graphene oxide (GO) (Figure 7b) [199]. The nanohybrid DES/GO-Fe_3_O_4_ exhibited good characteristics of organic dye absorbents. The study indicated that the conjugation probably occurred due to DES’s rendering of chemical bonds rather than physics interaction. Using DES as a functional medium, high uniformity and narrow size distribution of FeNPs in the nano-hybrids were obtained. Recently, the behavior absorbs layer of ionic surfactants (sodium dodecyl sulfate (SDS) and cetyltrimethylammonium bromide (CTAB) in DESs (ChCl-EG and ChCl-Gly) have been studied to indicate the new role of DESs in functionalization process [201]. In both as-prepared DESs, SDS was completely desorbed, whereas CTAB remains absorbed at positive and negative potentials. The stronger solvophobic interaction alkyl chains in DESs allowed low-concentrated CTAB self-assemble into a robust coating. Since different effects of surfactants on the electrochemical response of DES/electrode structure are explored, choosing a suitable surfactant is essential to ensure effective adsorption and desorption work. Typically, using DESs as functionalization agents through conjugation ligands supplier or new functional group addition improves the nanomaterials’ stability, dispersibility, and properties. The particular combination of DESs and additive surfactants can improve the materials’ target chemical and physical characteristics.

### 3.6. DES-Based Nano-Catalytic System

The nano-catalytic system-based DESs have been described. DES can be utilized to immobilize the nanostructures or support interactions of the catalytic system and enhance the catalytic activity. The first catalytic-based DES platform for organic transformation was reported by Zamani and coworkers [202]. The magnetic nanoparticle (MNPs) anchored DESs were used for catalysis. The reusable catalyst system included a DESs-based ChCl and ρ-toluene sulfonic acid (ρTSA) covalently adsorbed on MNPs with a size of 12 ± 2 nm. It was indicated that the bonding of ChCl of DES to the MNPs surface increased the catalytic activity of DES. Previously, ion-exchange resin Amberlyst 15 induced dehydration of fructose and sucrose in eight DES has been explored [203]. The eight prepared DESs differed by their HBA and HBD. Generally, a distinctly beneficial effect of coupling Aberlyst 15 with dicarboxylic acid-based DES was observed. The structure of DES components affected the reaction process to influence HBD. The ChCl-GA DES performed the best cooperation and balance between catalytic ability and stability. Recently, the combination of reline DES, gold nanoparticles (AuNPs), and Titanium oxide (TiO_2_) was developed as a new catalysis system [204]. The reline DES and AuNPs supported TiO_2_ nanoparticles were investigated with great prominence in various sections of oxidation for catalyzing reactions. Similarly, reline is combined with the magnetic nanocatalyst as a green and reusable solvent.

### 3.7. DES-Based Nanofluidic System

**Figure 8:**
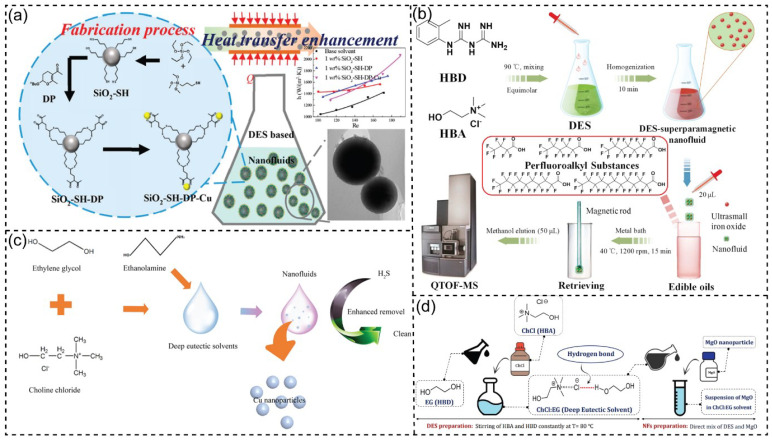
(**a**) Schematic representation of the silica-filled DES-based nanofluids for energy transportation. Reprinted with permission from ref. [214]. Copyright 2019 American Chemical Society. (**b**) Pretreatment procedure by using DES-based superparamagnetic nanofluid. Reprinted with permission from ref. [215]. Copyright 2021 Elsevier. (**c**) Scheme of utilizing of DES-based nanofluidic system and Cu nanoparticles for enhancing hydrogen sulfide removal. Reprinted with permission from ref. [216]. Copyright 2021 Elsevier. (**d**) Graphical illustration of the fabrication procedure of DES and design of DES-based nanofluids. Reprinted from ref. [220].

Recently, nanofluids have been applied for heat transfer applications where nanoparticles and ionic liquids (ILs)/DESs have been utilized [205,206,207,208]. In particular, DESs -a new generation of ILs- have been extensively investigated to prepare nanofluids, which are economical, eco-friendly, and biodegradable [23,209,210,211]. Besides, some common nanoparticles (e.g., copper, silica, magnesium oxides), carbon nanotubes (CNT), or graphene have been used for synthesizing nanofluids [212,213]. For example, Liu et al. suggested using a chemically decorated silica nanoparticle (SiO_2_) filled DES to improve the thermal conductivity and static stability of the synthesized nanofluids in energy transfer applications (Figure 8a) [214]. The silica nanoparticles were modified with copper, resulting in an enhanced thermal conductivity of silica nanoparticle (SiO_2_) filled DES nanofluids (13%). Walvekar and coworkers introduced the DES-carbon nanotube (CNT)-nanofluids by dispersing CNT into DESs using an ultrasonic technique without any stabilizer [208]. The authors found that the dispersion of CNT in DESs enhanced the thermal stability of DES-CNT-nanofluids, and these synthesized nanofluids had a lower freezing point and vapor pressure than ethylene glycol (EG) and triethylene glycol (TEG). Fan et al. described a superparamagnetic nanofluid based on a ChCl/1-(0-tolyl)biguanide DES for identifying perfluoroalkyl substances in edible oils (Figure 8b) [215]. For the microextraction of perfluoroalkyl substances (PFASs), the nanofluid acted as a sorbent material that exhibited high sensitivity (detection limit: 0.3–1.6 pg g^−1^) with a simple and rapid test for the enrichment and determination of trace PFASs. For H_2_S removal performances, a nanofluid system containing DESs and Cu nanoparticles was used with excellent outcomes for H_2_S removal (Figure 8c) [216]. The high regeneration performance of non-aqueous nanofluid systems was also observed. In addition, there are several studies on choline chloride and ethylene glycol–based DES for synthesized nanofluids for energy transportation [217,218]. Liu and coworkers used the combination of ChCl-EG DES as the based solvent and silica-decorated graphene for improving the stability of nanofluids, with 11.26% thermal conductivity enhancement [219]. Jafari et al. presented some new nanofluids based on DESs (Figure 8d) [220]. In this study, to prepare nanofluidics, four different DESs were used for dispersing MgO nanoparticles, and thermal conductivity was almost constant. Recently, DES ChCl-based nanofluids were formed by dispersion of nano-TiO_2_, Fe_2_O_3_, CuO, SiC, and carbon. These nanofluids exhibited an increase in thermal conductivity and heat capacity up to 4.23 and 26.23%, respectively [221]. In conclusion, DES-based nanofluids demonstrated potential prospects in the low-grade thermal energy utilization fields, specific heat capacity, thermal conductivity, and wide working temperature ranges.

## 4. Conclusions

This review summarizes the significant potential of DESs in nanomaterial fabrication. Currently, the publications and applications of DESs in chemical/electrical reactions have been growing fast, especially in nanostructure fabrication, due to their ability to overcome the challenges of conventional solvents, such as the hydrogen evolution issue of an aqueous solution. The considerable potential of DESs in fabricating various nanomaterials and nanomaterial-based systems has been studied; however, the use and design of DESs in developing renewable materials and nano-based systems such as microfluidic devices and chips are still limited and need to be explored to open up new horizons of new nanotechnology techniques and approaches. Additionally, the physiochemical properties of DESs can be controlled by fine-tuning HBAs and HBDs components. Their attributes of the high solubility of the polymer and other chemical substances allow DESs based fabrication strategies. They are applied in various roles, such as reaction media, catalysts, structure direction, morphology control, and assembly support, contributing to sustainable nanostructure engineering. This provides opportunities for nanotechnology to meet the circular economy, and a large amount of related research will be a breakthrough in the future.

## Data Availability

No new data were created or analyzed in this study. Data sharing is not applicable to this article.
